# A Review of miRNAs as Biomarkers and Effect of Dietary Modulation in Obesity Associated Cognitive Decline and Neurodegenerative Disorders

**DOI:** 10.3389/fnmol.2021.756499

**Published:** 2021-10-07

**Authors:** Maddie Perdoncin, Alec Konrad, Joshua R. Wyner, Samir Lohana, Sneha S. Pillai, Duane G. Pereira, Hari Vishal Lakhani, Komal Sodhi

**Affiliations:** Department of Surgery and Biomedical Sciences, Marshall University Joan C. Edwards School of Medicine, Huntington, WV, United States

**Keywords:** obesity, adipose tissue, cognitive decline, neurodegenerative disorders, microRNA

## Abstract

There has been a progressive increase in the prevalence of obesity and its comorbidities such as type 2 diabetes and cardiovascular diseases worldwide. Recent studies have suggested that the crosstalk between adipose tissue and central nervous system (CNS), through cellular mediators and signaling pathways, may causally link obesity with cognitive decline and give rise to neurodegenerative disorders. Several mechanisms have been proposed in obesity, including inflammation, oxidative stress, insulin resistance, altered lipid and cholesterol homeostasis, which may result in neuroinflammation, altered brain insulin signaling, amyloid-beta (Aβ) deposition and neuronal cell death. Since obesity is associated with functional and morphological alterations in the adipose tissues, the resulting peripheral immune response augments the development and progression of cognitive decline and increases susceptibility of neurodegenerative disorders, such as Alzheimer’s Disease (AD) and Parkinson’s Disease (PD). Studies have also elucidated an important role of high fat diet in the exacerbation of these clinical conditions. However, the underlying factors that propel and sustain this obesity associated cognitive decline and neurodegeneration, remains highly elusive. Moreover, the mechanisms linking these phenomena are not well-understood. The cumulative line of evidence have demonstrated an important role of microRNAs (miRNAs), a class of small non-coding RNAs that regulate gene expression and transcriptional changes, as biomarkers of pathophysiological conditions. Despite the lack of utility in current clinical practices, miRNAs have been shown to be highly specific and sensitive to the clinical condition being studied. Based on these observations, this review aims to assess the role of several miRNAs and aim to elucidate underlying mechanisms that link obesity with cognitive decline and neurodegenerative disorders. Furthermore, this review will also provide evidence for the effect of dietary modulation which can potentially ameliorate cognitive decline and neurodegenerative diseases associated with obesity.

## Introduction

Obesity has become a growing public health concern and it is estimated that by 2025, one out of every five adults will be obese worldwide (Mohammed et al., 2018). Furthermore, it is estimated that approximately 300,000 people die from obesity related causes every year (Mokdad et al., 2000). It has been approximated that before the year 2030, there will be a 30% increase in obesity prevalence and a 133% increase in the prevalence of severe obesity (Finkelstein et al., 2012). The current prevalence and an increase in the incidence of obesity will undoubtedly give rise to obesity associated comorbidities. Previous studies in a large cohort have demonstrated that body mass index (BMI) significantly correlates with risk of mortality, as well as the development of comorbidities such as type 2 diabetes and cardiovascular diseases (Berrington de Gonzalez et al., 2010).

Obesity is characterized by an increase in lipid accumulation and central adiposity, causing a significant risk of developing type 2 diabetes and cardiovascular disease (Pulgaron and Delamater, 2014; Tchang et al., 2021). The mechanism underlying this association is related to the dysregulation of glucose metabolism that results from lipotoxicity, consequently resulting in an increased insulin resistance or decreased insulin sensitivity (Kahn et al., 2006; Yazici and Sezer, 2017). Furthermore, cytokines secreted from adipose tissue, called adipokines, have been shown to be altered in obesity (Mattu and Randeva, 2013; Fasshauer and Bluher, 2015), which plays an important role in the development of adipose tissue induced inflammation and oxidative stress (Kahn et al., 2006). While these adipokines are under homeostatic control in normal physiological conditions, there is excessive production of reactive oxygen species (ROS) and dysregulation of inflammatory factors in an obese state which leads to a metabolic crisis (Kwon and Pessin, 2013). This inflammatory state and oxidant stress in obesity is mediated by dyslipidemia, insulin resistance and unmetabolized glucose substrate which leads to mitochondrial dysfunction (Barazzoni et al., 2018). It is also known that the ROS are generated through excessive substrates provided by a high calorie diet that directly impair insulin signaling in obesity (Rains and Jain, 2011). Apart from that, the dysregulation of adipokines and excessive generation of ROS has also been indicated to mediate the pathogenesis of cardiovascular diseases (Kwon and Pessin, 2013). It is also thought that obesity provides the environment necessary to mediate phenotypic changes in the adipocytes that result in macrophage recruitment and a subsequent increase in the macrophage to adipocyte ratio within the adipose tissue (Nakamura et al., 2014). Obesity has also been linked with localized hypoxia in adipose tissue through vascular changes (Ye, 2009; Nakamura et al., 2014).

The cumulative line of evidence shows that altered levels of adipokines as well as subsequent oxidative stress in obesity also has deleterious effects in the central nervous system (CNS) which has been linked to increased incidence of neurodegenerative disorders (Procaccini et al., 2016; Salim, 2017). Studies have demonstrated that midlife obesity was associated with an increased risk for the development of Alzheimer’s Disease (AD) (Ronnemaa et al., 2011), Parkinson’s Disease (PD) (Chen et al., 2004), and decline in cognitive function in the general population (Nguyen et al., 2014). Specifically, a large population based study showed that obesity at midlife, along with associated risk factors such as high cholesterol levels and high blood pressure, each almost doubled the risk for dementia with an even higher risk (odds ratio of 6.2) among the patient having all risk factors combined (Kivipelto et al., 2005). One of the pioneering epidemiological evidences, the Rotterdam Study, have shown that the risk of dementia and AD almost doubles in patients with type 2 diabetes (Ott et al., 1999). Interestingly, the Rotterdam study also showed that the risk of dementia and AD was significantly higher in patients with severe diabetes at baseline. A summary of clinical evidence from literature that suggests obesity is associated with cognitive decline and neurodegenerative disorders in illustrated in Table 1. Neurodegenerative diseases are characterized by toxic accumulation of proteins in the brain as well as consequential loss of neuronal cell number and function. Some commonly found proteins that accumulate in the brain are Aβ, prion protein, tau, α-synuclein, TARDBP, and FUS protein (Kovacs, 2017). The development and progression of neurodegenerative disorders relies primarily on the altered expression of these protein. The Aβ hypothesis is a well-established explanation for the deposition of Aβ in AD brains. The evidence of Aβ deposition alone is the first Alzheimer’s pathologic change of AD (Jack et al., 2018). Studies have shown that Aβ starts accumulating in brain regions such as precuneus, medial orbitofrontal and posterior cingulate cortices in pre-clinical AD patients (Palmqvist et al., 2017). Another important process in AD is associated with hyperphosphorylation of tau in neurofibrillary tangles, with new evidence from human AD brain samples demonstrating phosphorylation of Tau at multiple serine and tyrosine residues in different brain regions (Neddens et al., 2018). The cumulative line of evidence suggests that Aβ peptide for amyloid plaques and tau for neurofibrillary tangles work synergistically to alter neuronal function in AD, further demonstrating that Aβ triggers Tau pathology (Gotz et al., 2001; Lewis et al., 2001; Hurtado et al., 2010). While these studies suggest that Aβ functions upstream of tau, evidence shows that tau may also influences Aβ (Leroy et al., 2012). This raises the possibility that Aβ may trigger a pathological feedback loop with tau and characterize biological changes associated with AD (Bloom, 2014; Quiroz et al., 2018). The hyperphosphorylation of tau, along with aggregation of α-synuclein protein, is also known to play a mechanistic role in the development and progression of PD, as evidenced by murine models of parkinsonism (Duka et al., 2006; Haggerty et al., 2011). The synergistic action of tau and α-synuclein results in the formation of Lewy bodies, leading to the degeneration and loss of dopaminergic neurons, a hallmark in progression of PD (Haik et al., 2008). Further evidence from murine models suggests that aggregation of α-synuclein leads to nuclear factor erythroid 2–related factor 2 (Nrf2) deficiency that aggravate neuronal death in PD (Lastres-Becker et al., 2012). The overexpression of α-synuclein also contributes to the modulation of vascular pathology and causes blood brain barrier (BBB) leakage in PD (Elabi et al., 2021). Extensive research in murine models and clinical studies have shown that the aggregation of these toxic proteins, through their respective degenerative pathological processes, result in excessive production of inflammatory factors, such as TNFα, which are then transported across “leaky” BBB (Lastres-Becker et al., 2012; Maphis et al., 2015; Lee et al., 2021). These inflammatory factors alter and cross BBB through either active transport across BBB, through protein specific receptors on endothelial cells of BBB or passive transport via circumventricular organs, through simple diffusion (Pan et al., 2007; Fu, 2018). The transport of inflammatory factors, such as TNFα, across BBB allows these protein to be freely secreted in neuronal tissue, which induces neuroinflammation (Kim et al., 2012). In addition to harmful generation of ROS and other intermediates, adipokine dysfunction has also been indicated in the development of neuroinflammation, cognitive decline, and neurodegenerative diseases (Forny-Germano et al., 2018; Lee et al., 2019). Leptin, an important adipokine, produced primarily by adipose tissue, have been extensively shown to regulate hippocampal synaptic function (Dumon et al., 2018; McGregor et al., 2018), hippocampal neurogenesis (Garza et al., 2008) and neuronal morphology (O’Malley et al., 2007). Since adipokine leptin has free access to circumventricular organs, leptin is actively transported across BBB through leptin receptor (Di Spiezio et al., 2018; Harrison et al., 2019) and alters hippocampal function. Another important adipokine, adiponectin, have been shown to have neuroprotective effects in brain including regulation of hippocampal neurogenesis (Yau et al., 2014), neuronal excitability (Qiu et al., 2011), and protection from neuroinflammation (Chen et al., 2009; Chabry et al., 2015). While some studies suggest that adiponectin does not cross BBB (Pan et al., 2006; Spranger et al., 2006), contrary evidence suggests that adiponectin crosses BBB and induces neuronal alterations under diseased state (Qi et al., 2004; Yau et al., 2014). The adipokines, such as TNFα and interleukin 6 (IL6), that act as proinflammatory molecules are also able to cross the BBB and augment their function once localized to neuronal tissue (Banks et al., 1994; Pan et al., 1999; Pan and Kastin, 2002; Geng et al., 2018). It has even been proposed that these adipokines, that do not originate from neuronal tissue and enter the CNS by crossing the BBB, can work in unison with local factors that are produced by microglia within the brain to induce neuronal inflammation (Forny-Germano et al., 2018). Hence, these adipokines, such as adipokine, leptin, TNFα, IL6 and mediators of microglial activation also contribute to neuronal insulin resistance (Andre et al., 2017; Maldonado-Ruiz et al., 2017). Neurodegenerative states associated with neuronal protein deposition are also highly associated with obesity and comorbidities such as type 2 diabetes and cardiovascular disease due to increased amount of proinflammatory markers that inflict damage on the CNS (Forny-Germano et al., 2018; Surguchov, 2020). This indicates that there is a growing field to investigate new therapeutic targets and preventative measures for obesity associated with cognitive decline and neurodegeneration.

**TABLE 1:** Summary of clinical evidence from literature for obesity associated cognitive decline and neurodegeneration.

References	Study population	Key findings	Conclusion
Gustafson et al., 2003	Cohort of 392 non-demented Swedish adults (166 men and 226 women) were followed up for 18 years from age 70–88 years.	- During the 18 years 93 patients were diagnosed with dementia. - Women that developed dementia were overweight. - An increase of every 1.0 kg/m^2^ in body mass index (BMI) was associated with increased AD risk by 36%.	- Obesity is a risk factor for dementia, particularly AD in women.
Mueller et al., 2012	Cohort of 43 young adults (aged 20–41) divided in two groups: overweight/obese (27 individuals: 15 female and 12 male) and control (16 individuals: 6 female and 10 male).	- Overweight and obese young adult had significantly reduced gray matter which correlated with increased serum neuron-specific enolase (NSE) levels in hippocampal and cerebellar clusters. - Two cerebellum sub-regions that represent parts of the cerebellum associated with cognition were shown to be mainly impaired: Lobule VIIa and Crus 1	- Overweight/obesity contribute to the heightened vulnerability brain regions, that host cognitive function, exhibit to neurodegeneration in later life.
Raine et al., 2017	Cohort of 77 obese children were included in 9-month physical activity randomized controlled trial.	- Physical activity influenced changes in visceral adipose tissue: obese children that did not underwent physical activity had increase in visceral adipose tissue. - Reduction in visceral adipose tissue or fat mass was inversely proportional to cognitive performance.	- Reduced obesity and improved adiposity due to physical activity was associated with cognitive function in children.
Deckers et al., 2017	Cohort of 1,807 cognitively healthy individuals (aged 24–83) were followed up at 6- and 12 years. Among the cohort, 545 adults were obese, while 190 adults developed obesity during the study follow up.	- Obese individuals demonstrated a significant decline in memory, executive function and processing speed - Individuals that developed obesity during the follow up period had worse performance on executive functions.	- There is a strong association between obesity and cognitive decline which is further exacerbated by age.
Ma Y. et al., 2020	- Cohort of 6582 participants who were aged ≥ 50 years and were dementia-free at baseline (46% men and 54% women). All patients were divided into subgroups as normal weight, overweight and obese.	- Overall 453 participants developed dementia during the follow-up period of 15 years - Participants who were obese at baseline had an increased risk of dementia incidence, which was independent of age and sex. The risk of dementia was even higher in obese patients who had hypertension and diabetes. - Women with central obesity had a 39% greater risk of dementia; - Further comparison with patients having normal BMI, showed that obese and adults with higher waist circumference had 28% higher risk of dementia.	- Increased body weight or higher abdominal obesity is associated with increased incidence of dementia.
Amen et al., 2020	Cohort of 17,721 adults (mean age 40.8 ± 16.2 years) was divided into subgroups of underweight, normal weight, overweight, obese and morbidly obese.	- Strong relationship of overweight and obesity with brain hypoperfusion - Increased BMI was associated with significantly decreased cerebral perfusion as noted in brain scans. - Decreased cerebral perfusion was noted among all brain regions, including temporal lobe, parietal lobes, posterior cingulate, precuneus and hippocampus, that influence AD pathology.	- Increased BMI is strongly associated with decreased cerebral perfusion, hence obesity influences AD pathology.
Dake et al., 2021	Cohort of 172 participants in which neuroimaging was performed. Patients were categorized into mild AD, mild cognitive impairment and cognitively healthy.	-Obesity was significantly associated with reduced white matter integrity in brain and cerebral blood flow of temporo-parietal regions. - There was a direct correlation between obesity and gray matter volume.	-Obesity is associated with altered neural tissue in cognitively healthy and patients with mild cognitive impairment. -Normal BMI may prevent worsening of brain structure in mild AD patients.
Hassing et al., 2009	Cohort of 1152 participants aged between 45–65 years old, with up to 40 years follow up.	-During follow up period, 312 participants were diagnosed with dementia. -Participants with high BMI had 59% higher risk of developing dementia. -Statistical adjustment for diabetes and vascular disease increased the risk of vascular dementia. -The incidence of dementia, AD and vascular dementia was more common in women.	-The overweight status in midlife is a risk factor for the development of dementia, AD and vascular dementia in men and women.
Whitmer et al., 2008	Cohort of 6583 individuals with average follow up of 36 years.	-Dementia was diagnosed in 1049 individuals. -High sagittal abdominal diameter presented with almost threefold increased risk for the development of dementia. -Individuals who were obese having high BMI and high sagittal abdominal diameter showed the highest risk for development of dementia.	-Central obesity is a potent risk factor for the development of dementia.

*The table presents clinical evidence from studies with large patient cohorts along with the key findings from the study. Based on these key findings, there is conclusive evidence that suggests a strong association of obesity with cognitive decline and development of neurodegenerative disorders.*

Although obesity and neurodegeneration are distinct pathological conditions, there are important molecular markers of each that may link the two together, such as miRNAs. miRNAs are small, non-coding RNAs that help regulate gene expression by silencing or activating mRNA transcripts (O’Brien et al., 2018). While there is an extensive evidence demonstrating the mechanistic action of miRNAs in exacerbating obese phenotype (McGregor and Choi, 2011; Mentzel et al., 2015; Iacomino and Siani, 2017), as well as recent evidence in literature demonstrating the role of circulating levels of miRNAs in neurodegenerative disease (Sharma and Lu, 2018; Salama et al., 2019; Yuen et al., 2021). However, there have been limited research that links the underlying mechanisms operant with transcriptional regulation of miRNAs in cognitive decline and neurodegeneration caused by obesity and associated comorbidities. The study of miRNAs in obesity associated neurodegenerative disorders and their potential role as biomarkers has high translational applicability, since miRNAs are highly stable in various body fluids and the sequence of most miRNAs are conserved among different species (Lakhani et al., 2018). Furthermore, the expression of some miRNAs are specific to individual tissues and biological states (Lakhani et al., 2021). Hence, this review will study the role of several miRNAs and aim to elucidate underlying mechanisms that link obesity with neurodegenerative disorders in light of increased oxidative stress and dysfunctional adipokine signaling. These underlying mechanisms, associated with the transcriptional regulation of miRNAs, may allow for new therapeutic targets for obesity associated cognitive decline. In addition to investigating possible mechanisms using miRNA, this review will also provide evidence for effect of dietary modulation in potentially ameliorating cognitive decline and neurodegenerative diseases associated with obesity.

## Obesity Associated Cognitive Decline and Neurodegeneration

Obesity and associated metabolic disorders can affect not only metabolic homeostasis but also brain function, resulting in onset of non-genetic cognitive decline and neurodegenerative disorders (Lee et al., 2018; Graham et al., 2019). Recent studies have extensively investigated the anthropometric association of obesity and cognitive decline, however the mechanisms linking these pathological conditions remain highly elusive. One of the well-studied notions is the presence of oxidant stress caused by inflammation in obesity (Betteridge, 2000; Preiser, 2012). Previous studies have shown that mechanisms operant within adipocytes, under obese state, produce proinflammatory markers that are able to cross the BBB, resulting in oxidant stress which directly affect neuronal processes (Klein and Ackerman, 2003). Evidence suggests that these cytokines may cross BBB through active transport across BBB or through passive transport through areas in the brain where BBB in incomplete and the cytokines may cross through simple diffusion (Yarlagadda et al., 2009). Furthermore, studies have also shown that these inflammatory factors may also alter the BBB integrity by damaging the tight junctions of microvascular endothelial cells that form the BBB, resulting in an increased permeability (Yarlagadda et al., 2009). Mitochondrial dysfunction is thought to play a large role in the process of oxidative damage to the brain as well (Albers and Beal, 2000; Picca et al., 2020). Dysfunctional mitochondria produces ROS that interact with intermediates involved in neuronal processes and disturb normal homeostatic function of these cells (Onyango and Khan, 2006). Studies have extensively shown that excessive production of ROS damages mitochondrial DNA as well as causes lipid peroxidation and oxidative phosphorylation (Guo et al., 2013). The diet induced obesity significantly alters the expression of peroxisome proliferator-activated receptor gamma coactivator 1-alpha (PGC1α), a master regulator of mitochondrial biogenesis, also affecting the expression of its transcriptional complexes in hippocampus (Rius-Perez et al., 2020). Since, neuronal mitochondria regulates synaptic plasticity, the alteration of PGC1α expression directly affects hippocampal neuronal function (Cheng et al., 2012; Choi and Han, 2021). This obesity mediated release of inflammatory factors directly results in neuroinflammation, while subsequent oxidative damage caused by this inflammation may significantly damage the CNS, alter synaptic plasticity of the neurons, affect the development of neuronal synaptic activity and function and may lead to neuronal death (Khan and Hegde, 2020).

**FIGURE 1:**
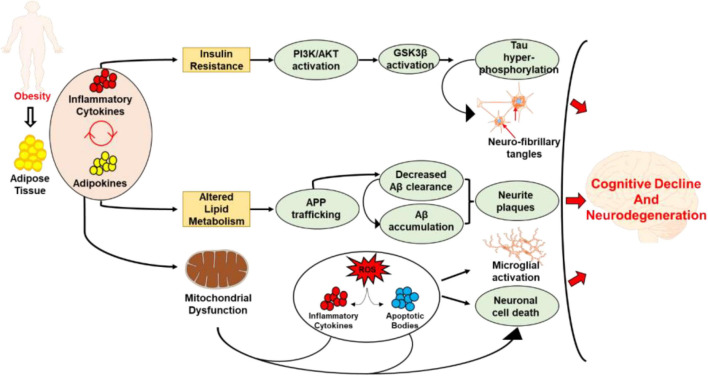
Schematic representation demonstrating the mechanisms related to obesity associated cognitive decline and neurodegeneration. The excess accumulation of adipose tissue and the resulting release of adipokines and inflammatory cytokines leads to insulin resistance, altered lipid metabolism, and mitochondrial dysfunction. While the insulin resistance leads to tau hyperphosphorylation and development of neurofibrillary tangles, altered lipid metabolism caused Aβ accumulation, and development of neurite plaques. On the other hand, mitochondrial dysfunction causes excessive production of ROS, inflammatory cytokines and apoptotic bodies which lead to neural cell death. The inflammation resulting from mitochondrial dysfunction also leads to microglial activation. Collectively, these processes lead to cognitive decline and the development and progression of neurodegenerative disorders.

Insulin resistance is also associated with diet induced obesity and frequently develops as a comorbidity of obesity (Castro et al., 2014). Studies have shown that impaired insulin signaling within insulin resistance directly affects the ability of neuronal glucose uptake (Holscher, 2020). This perturbance has been demonstrated to participate in the genesis of some neurodegenerative diseases due to the necessary role that insulin plays in providing neurons with glucose for metabolic processes (de la Monte, 2009; Femminella et al., 2021). The build-up of neuronal plaques and tau proteins within the brain is also potentiated by insulin resistance (Rad et al., 2018). This leads to devascularization within the cerebral cortex and cerebellum which can cause heightened levels of decline in cognitive function and the development of neurodegenerative diseases (Arnold et al., 2018; Rad et al., 2018). Since hippocampus plays an important role in regulating spatial learning and memory processes, several studies have shown obesity associated altered insulin signaling leads to altered hippocampal function. Specifically, studies have shown that diabetes induces structural and synaptic deficits in hippocampus which results in learning and memory deficits (Lee and Yau, 2020). Furthermore, under physiological condition of obesity and diabetes, murine models have been demonstrated to have significantly decreased cell proliferation, neuronal differentiation and cell survival in hippocampus (Ho et al., 2013). Subsequently, there is significant reduction in dendritic complexity and synaptogenesis in the hippocampus in obese and diabetic states, suggesting that hippocampal activity and function is highly affected by the metabolic imbalance caused by obesity (Ho et al., 2013). Studies have also demonstrated that insulin resistance leads to reduced phosphoinositide 3-kinase (PI3K) signaling and increased mitogen-activated protein kinase signaling, which results in decreased nitrogen oxide (NO) production (Boucher et al., 2014). The decrease in NO production has been shown to be associated with endothelial dysfunction. The altered production of NO in obesity and associated insulin resistance impairs neurovascular coupling which has been shown to cause cognitive impairment and neurodegeneration (Buie et al., 2019). Apart from that, obesity may also exacerbate the development and progression of cognitive decline and neurodegeneration through metabolic imbalance. Studies have shown that obesity may impair cognitive function through increased basal levels of corticosteroids and glucocorticoids. Furthermore, evidence from murine model suggests that microglial activation in diet-induced obesity is associated with reduction in dendritic spines and memory impairments (Bocarsly et al., 2015; Cope et al., 2018). Studies have also demonstrated that microglia plays an active role in synaptic degeneration when in a high calorie state (Cope et al., 2018). Neurogenesis and the maintenance of synaptic connections is also thought to be a targeted process in obesity. The cumulative line of evidence in murine models have demonstrated that diet induced obesity results in neurogenesis in the hippocampus which is directly correlated with the weight and adiposity (Bracke et al., 2019). A schematic representation of mechanisms operant is obesity associated cognitive decline is shown in Figure 1.

## miRNAs

From the perspective of neurodegeneration, the mechanistic insight into the impact of miRNA signaling have been investigated as significant biomarkers for AD diagnosis, disease prediction, prognosis and therapeutic purposes (Sharma and Lu, 2018; Zhao et al., 2020; Siedlecki-Wullich et al., 2021). The presence and persistence of various miRNAs in brain tissues and biofluids during neurodegenerative diseases suggest that miRNAs are involved in the regulation of multiple gene expression pathways throughout the human brain and CNS (Lukiw et al., 2012; Lukiw, 2020; Lukiw and Pogue, 2020; Ma F. et al., 2020). Detailed investigations based on fluorescent miRNA-array and RNA sequencing analysis, demonstrate that various miRNAs, and their enrichment in different sources of AD samples including patient blood serum, post-mortem brain tissues, etc. are highly sensitive to alterations in the biochemical, neurochemical, neuropathological, and/or cellular environment (Alexandrov et al., 2012; Lukiw, 2013a, b; Kumar and Reddy, 2020). As discussed in the previous section, growing evidences suggest that, obesity and other diet-induced metabolic dysfunctions can trigger and aggravate the development of neurodegenerative diseases (Ashrafian et al., 2013; Mazon et al., 2017; Lee and Yau, 2020). Hence, the exploration of some major miRNA, which are having possible role in obesity associated neurodegenerative disorders, may help to highlight perspectives of studying microRNA regulation in multiple brain signaling pathways.

### miR-26a

miR-26a is a well conserved miRNA whose overexpression or inhibition has been implicated in the pathogenesis of multiple disease, including obesity associated comorbidities such as reduced insulin sensitivity (Xu et al., 2020) and atherosclerosis (Feng et al., 2018). The cumulative line of evidence also suggests an important role of miR-26a in neurodegenerative disorders like AD (Liu et al., 2020) and PD (Su et al., 2019). Various mechanisms of gene regulation have been implicated in these diseased states, the pathophysiological consequences of which are exacerbated by the decreased expression of miR-26a. This suggests a potential link between low miR-26a levels and obesity associated cognitive decline and neurodegeneration.

Previous studies with *in vivo* models of obesity have demonstrated that reduced expression of miR-26a was associated with both insulin and glucose (Fu et al., 2015). Moreover, murine models of type 2 diabetes have shown that decreased expression of miR-26a was associated with decreased insulin sensitivity in pancreatic β cells (Xu et al., 2020). Further evidence from literature suggests that increased expression of miR-26a, decreases glucose stimulated insulin secretion (GSIS), thereby reducing hyperinsulinemia, a contributor in diabetes and obesity (Xu et al., 2020). GSIS is mediated in phases, relies on both actin remodeling for insulin granule release and focal adhesion, processes which are mediated by a focal adhesion kinase (FAK) and an extracellular signal-regulated kinase (ERK) (Xu et al., 2020). Hence, evidence suggests that an increase in circulating levels of miR-26a resulted in downregulation of phosphorylation of these kinases, resulting in less downstream effects and thus less GSIS (Xu et al., 2020). miR-26 family has been studied for regulating the human adipocyte differentiation by promoting characteristics of energy-dissipating thermogenic adipocytes (Karbiener et al., 2014). The potential role of miR-26a in the regulation of fatty acid and sterol metabolism has also been elucidated in experimental models of Non-alcoholic Fatty Liver Disease (Ali et al., 2018). Another study shows that miR-26a suppresses adipocyte progenitor differentiation and fat production by targeting Fbxl19 and highlights the critical role in regulating adipose tissue formation (Acharya et al., 2019).

The role of miR-26a have also been implicated in AD, which is a hallmark disease in the realm of neurodegeneration and the most leading form of dementia. Studies have shown that under expression of miR-26a is associated with the mechanisms underlying AD (Liu et al., 2020). By contrast, overexpression of miR-26a has been shown to be potentially neuroprotective. One mechanism behind the protective effect of miR-26a in AD is its downregulation of a target gene DYRK1A, which leads to decreased Tau phosphorylation and less Aβ build-up (Liu et al., 2020). This mechanism of upregulated miR-26a expression leading to less downstream phosphorylation is similar to the effect noted in obesity and GSIS in type 2 diabetes.

The common mechanisms of miR-26a and its effect on phosphorylation of Tau and kinases, have also been shown to play a similar role in PD. Studies in murine model of PD have shown a significant correlation between reduced expression of miR-26a and an increased expression of serine/threonine kinase called DAPK1 (Su et al., 2019). DAPK1 functions in part to phosphorylate alpha-synuclein, which is hallmark to Lewy Bodies, pathologic in PD (Su et al., 2019). By contrast, increased expression of miR-26a expression resulted in less DAPK1 expression (Su et al., 2019). Taken together, in both AD and PD, miR-26a expression and upregulation has been shown to use similar mechanisms to downregulate kinases which play a role in phosphorylation of pathological proteins in these diseases. The knockdown studies using cultured microglia demonstrate the role of miR-26a as a negative regulator of TLR4 triggered inflammatory response (Kumar et al., 2015). In addition, the miRNA expression profiling studies on the peripheral blood samples of patients with Amyotrophic lateral sclerosis and PD showed significant downregulation of miR-26a (Martins et al., 2011; Liguori et al., 2018). Furthermore, miR-26a has been included in a non-invasive miRNA-panel to improve the diagnosis of AD (Guo et al., 2017).

Apart from that, studies have also noted upregulation of miR-26a expression in budding neurites, and even higher expression in mature neurons, implicating its role in neural growth (Li and Sun, 2013). The underlying mechanism of this is suggested to be due to miR-26a downregulating PTEN, a molecule which suppresses neurite outgrowth (Li and Sun, 2013). Collectively, evidence from literature suggests a protective role of miR-26a and an association between low miR-26a expression and mechanisms underlying obesity associated neurodegenerative disorders (Absalon et al., 2013; Fu et al., 2015; Konovalova et al., 2019). This relays the importance of miR-26a as a critical marker, or even as a potential therapeutic target, in obesity associated cognitive decline and neurodegeneration.

### miR-29a

miR-29a is a member of the miR-29 family, which have been shown to target a diverse range of genes throughout the body (Kriegel et al., 2012; Wang et al., 2018). The involvement of miR-29a in several physiological and pathological processes has been extensively investigated, including its role in fibrosis (Harmanci et al., 2017), metastasis (Ngankeu et al., 2018), apoptosis (Wang et al., 2018), as well as a potential biomarker of non-alcoholic fatty liver disease (NAFLD) (Jampoka et al., 2018), highlighting its important role in metabolic dysfunction.

The role of miR-29a has been implicated in lipid metabolism and insulin resistance. A recent study has demonstrated the important role of miR-29a in fatty acid oxidation through regulation of PGC1α, an important marker of mitochondrial biogenesis (Cheng C.F. et al., 2018) which is intricately linked to obesity and type 2 diabetes (Liang and Ward, 2006; Massart et al., 2017). Overexpression of miR-29a was found to downregulate PGC1α, demonstrating miR-29a’s potential contribution to the development of metabolic disease (Massart et al., 2017). Apart from that, studies have also shown that miR-29a downregulates the expression of genes directly involved in fatty acid oxidation, including expression of pyruvate dehydrogenase kinase isoform (PDK4), fatty acid transport protein 2 (FATP2), and long chain specific acyl-CoA dehydrogenase (LCAD) (Wu et al., 2018).

In addition to lipid metabolism, miR-29a also plays a role in glucose homeostasis. One study found that increased levels of miR-29a in the anterior tibialis muscle of mice caused a decrease in glucose uptake and glycogen content, which was attributed to decreased levels of glucose transporter-4 (GLUT4) in the plasma membrane (Massart et al., 2017). The importance of miR-29a in glucose regulation have been further corroborated by other studies demonstrating that silencing of miR-29a expression increases insulin-induced glucose uptake in mice (Wu et al., 2018). This occurs through mechanistic action of miR-29a which downregulates peroxisome proliferator-activated receptor δ (PPARδ) (Wu et al., 2018) at the post-translational level (Zhou et al., 2016). PPARδ functions by increasing the transportation of GLUT4 to the plasma membrane of skeletal muscle (Wu et al., 2018), allowing for the uptake of glucose into cells. The role of PPARδ have also been implicated as a metabolic regulator and have been shown to enhance fatty acid catabolism, cardiac contractility, and lowers triglycerides (Barish et al., 2006). Together, these data suggest that miR-29a may play a role in impaired glucose uptake and obesity via downregulation of PPARδ. miR-29a mediated negative feedback loop regulation of peripheral glucocorticoid receptor signaling and its possible role in metabolic disorders such as insulin resistance, obesity and hyperglycemia has already been studied in detail (Glantschnig et al., 2019).

In addition to its role in metabolism, there is a cumulative line of evidence supporting miR-29a’s role in neurodegeneration. Specifically, expression of miR-29a have been shown to be increase by a factor of 2.2 in the CSF of patients with AD (Muller et al., 2016). Another study found that in the post-mortem human brain, miR-29a was upregulated in the anterior cingulate gyri of patients with PD (Goh et al., 2019). A recent study further demonstrated that miR-29a targets PGC1α and mitochondrial biogenesis and plays a significant role in maintaining neural cell function by preserving and promoting mitochondrial function (Li et al., 2017). Furthermore, expression of PPARδ, another target of miR-29a, has been found to be decreased in the brains of patients with AD (Strosznajder et al., 2021). This is not surprising because PPARδ is known to be a potent anti-inflammatory agent and acts to stabilize the myelin sheath as well as decreased Aβ deposits (Strosznajder et al., 2021). Other studies have also shown that a decline in the expression of PPARδ increases neuroinflammation, oxidative stress, and Aβ42 deposition, which highlights the protective role PPARδ serves in the brain (Strosznajder et al., 2021). The regulation of PPARδ and PGC1α through overexpression of miR-29a may serve as a potential mechanism for obesity associated neurodegenerative diseases. Microarray studies conducted on the frontal cortex derived from amyotrophic lateral sclerosis patients suggest that underexpression of miR-29a affects neurodegenerative processes by enhancing neuronal NAV3 expression in AD brains (Shioya et al., 2010). miRNA expression profiles of sporadic AD patients found that miR-29a is potentially involved in the regulation of APP and BACE1 gene expression which cause Aβ accumulation and appeared to be decreased in diseased brain (Hebert et al., 2008). miR-29a can also be a candidate biomarker for AD in cell-free CSF (Muller et al., 2016). The reverse transcription-quantitative real-time PCR studies followed by cloning and sequencing on blood serum of patients with PD also showed the significant reduction of miR-29a, which warrants further investigation of its potential serving as biomarkers for PD (Bai et al., 2017).

### miR-425

miR-425 is another miRNA that have been implicated not only in obesity related pathogenesis but also via various mechanisms in the brain and neurodegeneration (Olivo-Marston et al., 2014; de la Rocha et al., 2020). Although under-expression of miR-425 has a documented association with PD, evidence from literature suggests that overexpression of this miRNA is associated with multiple neurodegenerative disorders (Yuan et al., 2020). Furthermore, *in vivo* models of diet induced obesity have been shown to have increased inflammatory cytokine release and significantly increased expression of miR-425 (Olivo-Marston et al., 2014). Apart from that, miR-425 was also found to increase fat deposition and synthesis and have been shown to inhibit expression of proteins which play direct a role in negating adipogenesis (Qi et al., 2019). These lines of evidence suggest miR-425 overexpression as a possible culprit in obesity associated cognitive decline and neurodegeneration.

The upregulated expression of miR-425 have been supported by previous studies with *in vivo* models of diet induced obesity (Olivo-Marston et al., 2014). Studies in humans have found miR-425 levels to be significantly increased in diabetic patients versus control patients, providing further evidence that the expression of this miRNA has a substantial role in obesity (Luo et al., 2020). The underlying mechanism that supports this effect of miR-425 in diabetes suggests that it downregulates expression of monocarboxylate transporter 4 (MCT4), a lactate transporter, which when dysfunctional results in lactate build-up in endothelial cells, leading to apoptosis (Luo et al., 2020). Endothelial cell damage has been linked to diabetes, and as miR-425 expression has been shown to induce deleterious effects and endothelial cell death, there is clear indication of its role in obesity related disorders (Luo et al., 2020). Other studies in murine models of obesity revealed that miR-425 plays a direct role in the synthesis of fat, and that its inhibition prevents development and progression of obesity (Qi et al., 2019). This mechanism has been investigated and has revealed that miR-425 expression inhibits p38a, a gene that inhibits lipogenesis (Qi et al., 2019). miR-425 is closely associated with dysregulation of insulin/PI3K-AKT signaling in liver (Kwon et al., 2014). miR-425 is also reported to have an important role in the regulation of intestinal lipid metabolism (Ruiz-Roso et al., 2020).

In neurogenerative processes, upregulated expression of miR-425 was found in AD patients, which corresponded with increased tau phosphorylation in these patients (Yuan et al., 2020). *In vivo* models suggest the causative mechanism to be upregulation of miR-425 leading to decreased expression of heat shock protein 8, and as a result, increased tau phosphorylation at multiple residues and apoptosis of neural cells (Yuan et al., 2020). In other processes in the brain, miR-425 expression at basal and high levels have been shown to promote phosphorylated forms of AKT survival pathway in glioblastoma multiforme, revealing its role in neural related pathways (de la Rocha et al., 2020). Glioblastoma multiforme is well documented to cause cognitive deficits and neurodegeneration (Hanif et al., 2017; de la Rocha et al., 2020). The inhibition of miR-425 in these mice subsequently resulted in decreased phosphorylation of this pathway, and less brain tumor survival (de la Rocha et al., 2020). miR-425 deficiency triggers necroptosis of dopaminergic neurons in PD and the study propose miR-425 supplements as a probable therapeutic approach for neurodegenerative disease with neuron loss (Hu et al., 2019). Also another study identifies miR-425 as a biomarkers to discriminate mild Traumatic brain injury from severe (Di Pietro et al., 2017). Hence, miR-425 have a potential role in obesity and neurodegenerative disorders and may serve as potential biomarker of this clinical condition.

### miR-33

miR-33 is a family of miRNA that is well known to regulate lipid metabolism, cholesterol homeostasis, and fatty acid metabolism as well (Cirera-Salinas et al., 2012). While these processes may be involved more directly in obesity, emerging studies have begun to reveal the function that miR-33 has to play within neurodegeneration as well.

Studies have shown that the antagonism of miR-33 induces plaque regression and an increase in activity of the reverse cholesterol transport pathway in murine model of obesity (Rayner et al., 2011). The mechanism that miR-33 controls the regulation of cholesterol is through downregulation of the protein ATP-binding cassette transporter protein 1 (ABCA1), which is involved in the production of high-density lipoproteins (HDL) (Medvinskii et al., 1990). A proposed model states that ABCA1 assists in the migration of phospholipids from the cytoplasm to the extracellular fluid, or cholesterol efflux, and the binding of apoA1 to the plasma membrane of ABCA1-expressing cells to create nascent HDL (Wang and Smith, 2014). This is related to the topic at hand because lower HDL levels are associated with obesity (Rashid and Genest, 2007). miR-33 is involved in the down regulation of several genes encoding key enzymes involved in cholesterol efflux, fatty acid metabolism, and insulin signaling (Goedeke et al., 2013). These studies suggest that miR-33 plays a role in obesity through the regulation of cholesterol. Report shows that the genetic ablation of miR-33 increases food intake, enhances adipose tissue expansion, and promotes obesity and insulin resistance (Price et al., 2018). In addition, a study using mouse model of diet-induced obesity have shown that the therapeutic inhibition of miR-33 may promote whole-body oxidative metabolism but does not affect metabolic dysregulation (Karunakaran et al., 2015).

The role of miR-33 has also been implicated in the neurodegenerative processes. Studies have demonstrated that the knockdown of miR-33 improved synaptic plasticity, reduced inflammation, oxidative stress, and cell apoptosis (Wang et al., 2020). The direct intracerebral delivery of a miR-33 antisense oligonucleotide into mouse brain was shown to be an effective strategy for increasing brain ABCA1 expression/activity, which can have beneficial effects on various neurodegenerative and cardiovascular diseases (Jan et al., 2015). Evidence have also shown that miR-33 in the brain contributes to the maintenance of adaptive thermogenesis and whole-body metabolism via enhanced sympathetic nerve tone through suppressing GABAergic inhibitory neurotransmission (Horie et al., 2021). A proposed mechanism by which miR-33 regulates neurodegeneration is through the inhibition of ABCA1, a protein that is downregulated by the presence of miR-33. Accordingly, mice that were deficient in miR-33 expressed higher levels of ABCA1 in the brain (Kim J. et al., 2015). Furthermore, one particular study demonstrated that cells expressing high levels of miR-33 demonstrated higher levels of Aβ, a distinctive feature of AD, and lower levels of ABCA1 (Jaouen and Gascon, 2016), further supporting the argument that miR-33 is involved with the aggregation of Aβ through cholesterol homeostasis (Jaouen and Gascon, 2016). Accordingly, elevated levels of cholesterol have been found to increase the likelihood of developing AD as well (Shepardson et al., 2011), due to excessive accumulation of Aβ in the brain, which can lead to a disruption in neuronal function (Huang and Liu, 2020). It is suggested that altered cholesterol homeostasis through ABCA1, downregulated by the expression of miR-33, may lead to an increase in APP cleavage, thus causing an increase of Aβ in the brain (Montesinos et al., 2020). Aβ, in turn, propagates hyperphosphorylation of tau proteins, which can cause the aggregation of this protein and lead to the disruption of synapses. The mechanism of tau hyperphosphorylation and an excessive accumulation of Aβ is associated with the activation of the enzyme GSK3β (Huang and Liu, 2020). Hence, the potential role of miR-33 in altering cholesterol homeostasis and excessive Aβ accumulation may implicate a causal mechanistic link in obesity associated neurodegenerative disorder.

### miR-21

miRNA-21 has been known to be linked to the physiology of cancer, inflammation and cardiac disease (Sheedy, 2015; Yang et al., 2018). However, recent evidence from literature suggests its potential role in both the promotion of obesity as well as neurodegeneration. The overexpression of miR-21 has been shown *in vitro* to promote adipocyte differentiation through adipogenesis (Kang et al., 2013). Furthermore, *in vivo* models of miR-21 knockdown mice exhibited a reduction in obesity and an increase in weight loss (Seeger et al., 2014), suggesting that higher expressions of miR-21 may be correlated with obesity and weight gain. Additionally, levels of phosphatase and tensin homolog (PTEN) were restored to a in miR-21 deficient mice (Seeger et al., 2014). The role of PTEN have been implicated as a tumor suppressor (Hopkins et al., 2014), but has implications within cognitive decline (Zhang et al. 2006b) as well as insulin resistance (Pal et al., 2012). One particular study found that carriers of mutated PTEN were more overweight and had higher insulin resistance than control subjects (Pal et al., 2012). This is most likely due to the fact that it prevents the effect of the PI3K-AKT pathway, which is involved in insulin signaling (Pal et al., 2012). This pathway is also crucial in the normal function of β-cells, which produce insulin (Elghazi and Bernal-Mizrachi, 2009). Additionally, mice that had higher levels of PTEN expression had an increase in the use of energy and less prevalence of adiposity (Seeger et al., 2014), suggesting that the repression of this protein could cause an increase in the chance of developing obesity. Hence, overexpression of miR-21 and subsequent decrease in the levels of PTEN may promote obesity associated insulin resistance. The progressive increase in the expression of miR-21 in the serum samples of a study population with obesity and diabetes with or without fatty infiltration, shows its predictive efficacy as a biomarker (Pillai et al., 2020).

The role of miR-21 has also been implicated in neurodegenerative processes. Studies in AD patients, have shown that the expression of miR-21 was significantly upregulated as compared to control subjects (Giuliani et al., 2021). MicroRNA 21 is reported to be up-regulated in adipose tissue of obese diabetic subjects undergoing bariatric surgery (Guglielmi et al., 2017). The effect of miR-21 has been reported against ischemic neuronal death, possibly by targeting a tumor necrosis factor family cell death-inducing ligand (Fas ligand gene) (Buller et al., 2010). The regulatory effect of miR-21 on α-synuclein expression in neurons has been exploited as a protective mechanism of some drugs against Parkinson’s disease (Buller et al., 2010). Furthermore, a deficiency or mutation in PTEN, a target of miR-21, may cause aggregation of tau protein (Zhang et al. 2006b), which is a fundamental component of AD disrupting neuronal function by forming tangles (Gendron and Petrucelli, 2009). This evidence suggests that miR-21 can inhibit the expression of PTEN, thus causing the aggregation of tau. Additionally, PTEN may have an effect in the phosphatidylinositol 3,4,5 trisphosphate (PIP3) survival signaling pathway, which is involved in the exacerbation of tau toxicity (Zhang et al. 2006b). Hence, the role of miR-21 through regulation of PTEN may provide a mechanistic link in obesity associated neurodegeneration can may be a crucial biomarker for this condition.

### miR-34a

As a member of the miR-34 family of microRNAs, miR-34a has been extensively investigated in a number of biological processes including cell development, metabolism, and differentiation (Imani et al., 2018). Most recently, miR-34a has been found to be upregulated in both metabolic and neurodegenerative disorders. Specifically, one study found that miR-34a was one of the most highly elevated genes during the development of diet induced obesity (Pan et al., 2019). Furthermore, the study demonstrated that high levels of miR-34a in adipose tissue increased obesity-induced inflammation by regulating Krüppel-like factor 4 (klf4). The gene klf4, which is responsible for polarization of anti-inflammatory M2 macrophages, was found to be significantly decreased in the adipose tissue of obese individuals (Pan et al., 2019). Because miR-34a significantly represses klf4, mice with increased miR-34a expression experienced significantly increased transition to the pro-inflammatory M1 macrophage type, exacerbating the systemic inflammation and metabolic dysregulation, strongly associated with obesity (Pan et al., 2019). Evidence from other studies suggests that miR-34a directly targets and downregulates Sirtuin1 (SIRT1), an NAD + -dependent deacetylase, known to be protective against obesity (Choi et al., 2013). An important location of SIRT1 is in the hypothalamus, where it plays a vital role in regulating energy homeostasis and suppressing metabolic disease such as obesity and type 2 diabetes (Xu et al., 2018). Expression of SIRT1 stimulates mitochondrial biogenesis, fatty acid oxidation, and ketogenesis (Xu et al., 2018), further demonstrating the importance of miR-34a in the regulation of obesity associated metabolic processes. A study conducted in the plasma samples of obese children and adolescents showed that miR-34a is associated with insulin and HOMA-IR and thus have significant role in IR (Ahmadpour et al., 2018). A significantly upregulated expression of miR-34a has been observed in patients with obesity, diabetes and fatty infiltration, suggesting the possible role as a biomarker for disease progression (Pillai et al., 2020).

A recent study has also demonstrated the highly beneficial role SIRT1 plays in the brain, protecting against neurological disorders and cerebral ischemia (Xu et al., 2018). It was found that SIRT1 is expressed at low levels in the brains of patients with AD, where it acts to deacetylate AD-affected neurons causing repression of p53 and inhibition of apoptosis (Manjula et al., 2020). The levels of SIRT1 were also highly correlated to Braak stage, a system used to determine the severity of dysfunction in neurological pathologies (Burke et al., 2008; Manjula et al., 2020). Further, elevated levels of SIRT1 were shown to provide protection against the development of Huntington’s Disease 30532738 (Xu et al., 2018). The regulation of SIRT1 by miR-34a serves as a potential mechanism for the neurological dysfunction observed in these patients. In addition to SIRT1, the gene klf4 was also found to be protective against the development of cerebral injury 30297986 (Cheng Z. et al., 2018). Specifically, it was found that klf4 is involved in the processes of anti-inflammation, anti-apoptosis, and axon regeneration in the brain via the JAK-STAT3 pathway (Cheng Z. et al., 2018). The study performed for the microRNA abundance in CSF and brain tissue-derived extracellular fluid (ECF) from AD neocortex also showed dysregulation in the expression of miR-34a along with some other microRNAs (Alexandrov et al., 2012; Zhao et al., 2020). The differential profile of circulating miR−34a has also been explored in PD patients in comparison with age-matched control subjects (Grossi et al., 2021). Evidence shows that miR-34a-mediated autophagy, abnormal mitochondrial dynamics and SIRT1/mTOR signaling pathway has important role in the functional complications of brain associated with AD (Kou et al., 2017; Chen et al., 2019).

There is a cumulative line of evidence supporting the role of miR-34a in neurodegeneration. A recent study found that overexpression of miR-34a resulted in rapid cognitive decline in conjunction with intracellular Aβ deposits and tau hyperphosphorylation in mice (Sarkar et al., 2019). Furthermore, evidence suggests that overexpression of miR-34a downregulates the expression of neuroprotective proteins including SIRT1, ADAM10, and NMDAR2B (Sarkar et al., 2019). Another study demonstrated that miR-34a elevation is linked to numerous neurodegenerative processes including hearing loss and age-related macular degeneration (Chua and Tang, 2019). It was found that miR-34a significantly contributes to neuronal cell death during epileptic seizures and ischemic stroke via its regulation of downstream targets such as SIRT1 (Chua and Tang, 2019). Regulation of the genes SIRT1 and klf4 may serve as a mechanism through which miR-34a promotes obesity associated neurodegenerative disorders, implicating its importance as a biomarker.

### miR-200

The miR-200 family of micro-RNAs consists of miR-200a, 200b, 200c, 141, and 429 (Humphries and Yang, 2015). The family has been well-studied in cancer initiation and metastasis (Humphries and Yang, 2015), while some of its members have been implicated in metabolic (Belgardt et al., 2015) and neurological dysfunction (Fu et al., 2019). Evidence suggests that overexpression of the miR-200 family in mice induces pancreatic beta cell apoptosis, subsequently decreasing insulin production and inducing lethal type 2 diabetes (Belgardt et al., 2015; Magenta et al., 2017). Furthermore, these miRNAs induce apoptosis through positive regulation of the tumor suppressor Trp53 (Belgardt et al., 2015). The study in murine model of diabetes showed that miR-200 levels were elevated in the pancreatic islets of diabetic mice (Belgardt et al., 2015), further demonstrating the significance of the miR-200 family in metabolism.

In addition to inducing apoptosis, another study found that the miR-200 family is involved in metabolic processes through promoting insulin resistance. Mechanistically, miR-200 acts by targeting Zinc Finger Protein Family Member 2 (FOG2), a transcription factor that blocks insulin signaling. The miR-200 family also downregulates insulin receptor substrate-2 (IRS2), an important regulator of hypothalamic leptin and peripheral insulin sensitivity (Brady, 2004; Dong et al., 2016; Magenta et al., 2017). Silencing of mir-200a resulted in increased expression of leptin receptor and IRS2, decreased weight gain, and increased responsiveness to insulin in the liver (Crepin et al., 2014). Another mechanism through which the miR-200 family is involved in metabolic dysfunction is through promoting inflammation (Reddy et al., 2012). Both miR-200b and miR-200c have been found to downregulate ZEB1, contributing to the pro-inflammatory state associated with obesity (Reddy et al., 2012; Magenta et al., 2017). It was also found that miR-200c directly downregulates SIRT1 (Magenta et al., 2017), which has a significant role in protection from obesity and associated comorbidities (Mariani et al., 2018). A study conducted in the serum samples from obese preschoolers (aged 2–6 years) identified miR-200 as a s biomarkers of insulin resistance in obese condition (Masotti et al., 2017). Evidence shows that, miR-200c exerts its pathophysiological effects of obesity on endothelial function via oxidative stress (Ait-Aissa et al., 2020).

There is a cumulative line of evidence demonstrating the role of the mir-200 family in neurodegeneration. Similar to the role of miR-200 family in regulating SIRT1 in metabolic disease, it also plays a role in the regulation of SIRT1 in cognitive decline. Studies have shown that miR-200a was upregulated in AD and caused Aβ-induced neuronal apoptosis through downregulation of SIRT1 (Zhang et al., 2017; Fu et al., 2019). Additionally, the role that the miR-200 family plays in inducing inflammation in obesity through regulation of ZEB1 may also serve as a mechanism for inducing inflammation in neurodegenerative disorders. A large line of evidence supports enhanced inflammatory responses in the development of many neurological disorders including PD (Stojkovska et al., 2015) and Huntington’s disease (Valadao et al., 2020). The protein ZEB1 has been found to play a protective role in the brain, specifically against cerebral ischemia (Bui et al., 2009). Just as miR-200b and miR-200c downregulate ZEB1 in obesity, they may also play a role in its regulation during periods of ischemia. Another study reported that the miR-200 family is involved in multiple pathological processes including Aβ secretion, alpha-synuclein aggregation, and DNA repair (Fu et al., 2019). The expression studies of miR-200c in the serum of both AD mice and human AD patients demonstrate the role of miR-200c in the neuronal cell-intrinsic adaptive machinery, neuronal survival, and differentiation in response to Aβ induced ER-stress (Wu et al., 2016). miR-200b/c is reported to have role in reducing Aβ secretion and Aβ-induced cognitive impairment by promoting insulin signaling (Higaki et al., 2018). Furthermore, it has been established that miR-200 family along with other miRNAs can modulate energy homeostasis and body fat content by affecting leptin transduction pathway in pro-opiomelanocortin, Agouti-related peptide/Neuropeptide Y and melanocortin receptors-expressing neurons (Derghal et al., 2017). Given its significant roles in metabolism and cognition, the miR-200 family may serve as an important biomarker for obesity-associated neurodegeneration.

### miR-144

The role of miR-144 have been implicated in multiple pathologies, including its role as a tumor suppressor (Kooshkaki et al., 2020) and in erythropoiesis (Rasmussen et al., 2010). However, evidence from literature have also linked this miRNA to obesity as well as neurodegeneration. Recent studies have showed that miR-144 was overexpressed in the pancreas, adipose tissue, and liver of diabetic rats, further targeting insulin receptor substrate 1 (IRS1), which is crucial in insulin signaling (Patterson, 1990). Evidence have also shown that expression of miR-144 was upregulated in the adipose tissue of obese mice (Shen et al., 2018). The overexpression of miR-144 increased lipid accumulation and promoted adipogenesis while levels of CCAAT-enhancer binding protein alpha (C/EBPα) were also upregulated in the murine model of obesity. This is relevant because C/EBPα is known to promote adipogenesis and is also involved in positively regulating PPARγ, a protein involved in glucose metabolism (Shen et al., 2018). Other studies have also supported the role of miR-144 in regulating levels of C/EBPα suggesting a positive correlation (Chen et al., 2020). Additionally, it has also been demonstrated that miR-144 downregulates hydroxymethylglutaryl-CoA reductase (hmgcr), cholesterol 7a-monooxygenase A1 (cyp7a1), and ABCA1 in a model of diet induced obesity (Chen et al., 2020). These proteins have been shown to be involved in the regulation of cholesterol homeostasis (Chen et al., 2020), suggesting the role of miR-144 in cholesterol metabolism (Chen et al., 2020). In addition, levels of A Disintegrin and metalloproteinase domain-containing protein 10 (ADAM10), a target of miR-144 (Sun et al., 2017), have been demonstrated to be significantly decreased in obese subjects and which was associated with an increase an oxidative stress (Elsworthy and Aldred, 2020). The studies on the liver biopsies from morbidly obese subjects undergoing bariatric surgery showed that miR-33a/144 and their target gene ABCA1 may contribute to the pathogenesis of NASH in morbidly obese subjects (Vega-Badillo et al., 2016). Recently, a novel mechanism of miR-144 action in modulating the activity of Nrf2 through fumarate production, has been demonstrated for the impaired antioxidant response in obesity (Azzimato et al., 2021).

Apart from the role of miR-144 in obesity and associated metabolic dysregulation, studies have shown that miR-144 was upregulated in the cortex and cerebellum of patients with AD (Persengiev et al., 2011). Another study found that miR-144 downregulate the levels of ADAM10, inhibiting its protective effects against Aβ accumulation in AD (Cheng et al., 2013). On the other hand, the expression of miR-144 significantly decreased the levels of C/EBPα, in a model of Huntington Disease (Chiang et al., 2007). Additionally, the suppression of C/EBPα caused a decrease in the activity of the urea cycle, a hallmark of Huntington’s Disease (Chiang et al., 2007). A qRT-PCR study conducted using the brain samples of postmortem PD patients showed that downregulation of miR-144 in the brain is related to PD via activation of NF-κB signaling (Xing et al., 2020). miR-144 has been identified as a nuclear respiratory factor 2 (Nrf2)-regulating miR, which is reported to have role in responsiveness to insulin and insulin-like growth factor stimulation, which will have possible effect on brain glucose utilization in AD (Karolina et al., 2011; Paladino et al., 2018). Along with the inhibition of APP, miR-144 increased the expression of key genes involved in maintaining mitochondrial function, including peroxisome proliferator-activated receptor γ coactivator-1α (PGC-1α), nuclear respiratory factor 1 (Nrf-1) and mitochondrial transcription factor A (TFAM) in SH-SY5Y cells (Li et al., 2016). These evidence may further explore the importance of miR-144 in connection with metabolic function and neurodegenerative diseases including PD. In addition, the involvement of miR-144 in inducing cholinergic degeneration by impairing the maturation of nerve growth factor in Alzheimer’s Disease has been revealed in experimental models (Zhou et al., 2021). Although more research is necessary to understand the role of miR-144, evidence from literature provides a mechanistic link of miR-144 in obesity associated neurodegenerative disorders.

## Effect of Dietary Modulation in Obesity Associated Cognitive Decline and Neurodegeneration

This review highlights the role of several miRNAs that have been in implicated in both obesity and cognitive decline. While there is room for exploration of developing therapies targeted to these specific miRNAs in obesity associated cognitive decline, such model requires additional research and testing. However, one therapeutic approach that is currently available in the fight against obesity associated cognitive decline is preventing its development and progression through dietary intervention. As obesity and associated comorbidities have been linked to cognitive decline and neurodegenerative disorders, there is growing evidence that supports the idea that dietary measures such as fat reduction and calorie restriction can lead to prevention of obesity and also neuroprotective effects (Schafer et al., 2015; Tan and Norhaizan, 2019).

A high fat diet has been shown to lead to obesity and related health consequences such as insulin resistance, which have now been demonstrated to cause insulin resistance in the brain as well, playing a role in neurodegeneration (Kothari et al., 2017). Insulin resistance in the body has been shown to lead to metabolic syndrome, which is associated with multiple obesity associated pathologies such as cardiovascular disease and NAFLD (Kothari et al., 2017). A previous study have demonstrated decreased tyrosine phosphorylation and increased serine phosphorylation of insulin receptor substrate 1 (IRS1) in the whole brain tissues of mice fed a high fat and high sugar diet, resulting in neuronal insulin resistance and an increased production of inflammatory factors in the brain (Kothari et al., 2017). The study also observed increased levels of pSer^473^, associated with AKT inhibition and peripheral insulin resistance, in the whole brain tissue of these high fat diet fed mice. Evidence from *in vivo* models have also suggested that high fat diets increase the neuroinflammatory markers which play a role in hippocampal deficits in memory loss (Duffy et al., 2019). Other studies supported these results, showing high fat fed mice had consistently increased weight compared to those which were chow fed, and also that offspring of high fat fed mice had a statistically significant decline in memory exercises like maze escapes compared to the offspring of those on chow fed diets (Cordner et al., 2019). Moreover, the offspring of the high fat fed mice also showed differential expression of genes within the hippocampus, including significantly decreased expression of INSR, a gene which plays a role in insulin signaling and is thought to be involved in neuron maturation (Cordner et al., 2019). All these results taken together should provide meaningful evidence to link high fat diets not only to obesity, but also to the processes that modulate cognitive decline and impaired neuronal function.

Therapeutic measures that have been shown to prevent obesity associated disorders and diseases of cognitive decline includes implementation of Mediterranean diet and caloric restriction (Schafer et al., 2015; van den Brink et al., 2019). Mediterranean diet comprises of a higher than usual fruit and vegetable intake, increased olive oil usage as the hallmark fat included, and otherwise decreased fat intake by limiting items like processed and red meat (McGrattan et al., 2019). This diet has shown antioxidant properties and an array of decreased proinflammatory factors such as decreases in IL-6, IL-1, and TNF-alpha, all factors which mediate inflammation (McGrattan et al., 2019). These inflammatory markers, especially IL-6 have been positively linked with neurodegeneration, decreased cognitive decline, and decreased brain volume (McGrattan et al., 2019). In patients with metabolic syndrome, a condition interconnected to obesity, starting on a Mediterranean diet resulted in decreased in pro-inflammatory markers like IL-6 (McGrattan et al., 2019). Studies with murine models fed with Mediterranean diet had elevated levels of polyphenol, which come in most part from the olive oil, hallmark to the Mediterranean diet (Reutzel et al., 2018). These mice were found to have increased ATP levels in the brain, along with improved cognitive function in memory tasks compared to their control counterparts not fed with polyphenol heavy Mediterranean diet (Reutzel et al., 2018). Another therapeutic approach worthy of consideration is that of caloric restriction. Studies in mice models predisposed to Aβ plaque build-up, characteristic of AD, revealed that a caloric restriction of even 30% resulted in downregulation of complex required for cleavage of APP and subsequent build-up of Aβ protein in mice (Schafer et al., 2015). These results show that calorie restriction as a means of dietary intervention can potentially play a role in protection against neurodegeneration and cognitive decline as it can result in reduced Aβ. Caloric restriction has been clearly shown to play a role in prevention of obesity and weight loss as well, and so its role as a potentially preventative therapy against obesity associated cognitive decline should not go understated (Zubrzycki et al., 2018). Taken all together, the limited intake of high fat diets as well as caloric restriction and a dietary focus on the foods in the Mediterranean diet could provide protection against obesity and associated cognitive decline and neurodegenerative disorders.

## Conclusion

**FIGURE 2:**
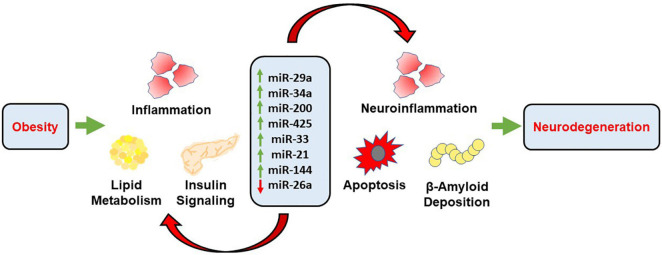
Schematic representation demonstrating the mechanistic role of miRNAs in obesity associated neurodegeneration. Obesity propagates inflammation, lipid accumulation and alters insulin signaling through the transcriptional role of respective miRNAs. These miRNAs further mediate neuroinflammation, apoptosis and accumulation of Aβ through distinct pathways, along with alterations associated with obesity which further results in cognitive decline and neurodegeneration.

This review aims to highlight the mechanistic role of eight microRNAs involved in the pathogenic link between obesity and its associated comorbidities, such as type 2 diabetes and cardiovascular disease, and neurodegenerative disorders characterized by cognitive decline. It has been demonstrated that obesity is characterized with increased levels of oxidative stress and inflammation that is the direct result of increased lipid accumulation. This state of metabolic dysregulation favors pathophysiological mechanisms that may lead to CNS neurodegeneration and cognitive decline (Fischer and Maier, 2015). These pathophysiological mechanisms propagate the development of neurodegenerative diseases through the generation of oxidative stress and inflammation that is damaging to neuronal tissue. There may be a myriad of mechanisms, but the one this review aims to isolate and further identify is through the generation of aberrant levels of miRNAs involved in key cellular functions. Some of these functions are glucose and lipid metabolism, molecular signaling pathways, and apoptotic pathways. When these functions are perturbed, it can lead to further development of oxidative stress, inflammation, and apoptosis (Figure 2). This continual cycle promotes the further development of pro-inflammatory markers that wreak cellular havoc on physiologically homeostatic systems. We also investigated the efficacy of dietary interventions on the disruption of these cyclic pathways that favor the pro-inflammatory state. As a non-invasive and inexpensive remedy, dietary intervention plays an important role in the amelioration of the obese-phenotype and development of its associated comorbidities. The regression and termination of the development of the obese phenotype through dietary interventions may even have important ramifications in light of CNS pathologies that are directly linked to the obese state. It is thought that the modification of diet can slow pro-inflammatory pathways to the point that the CNS is not receiving any harmful overflow that may lead to the development of neurodegenerative associated cognitive decline.

The microRNAs that were investigated showed insight into the development and continuation of pro-inflammatory states that favor oxidative stress, and thus may be linked with the severity of neurodegenerative diseases affiliated with the obese phenotype. The role of these microRNAs is variable, with involvement in a few pathways rather than widespread involvement with minimal role in several pathways. One of the roles is the inhibition of glucose metabolism when levels of the corresponding miRNA are perturbed. The microRNAs that are thought to have involvement in this are miR-29a, miR-200 family, miR-26a, miR-21, and miR-144. The contribution to the disruption of glucose metabolism is variable between each miRNA, but the basis for disruption includes some involvement in either insulin resistance or metabolic glucose pathways (Ono, 2011; Williams and Mitchell, 2012). Another way that miRNAs encourage the development and continuation of the obese phenotype associated with neurodegeneration is through the disruption of lipid metabolism. The role of miR-29a, miR-33, miR-21, and miR-144 have been implicated in lipogenic pathways as well as cholesterol metabolism. Furthermore, another major pathway that plays an important role in the development of neurodegenerative diseases associated with the obese state is the propagation of pro-inflammatory markers, the mechanism of which are targeted by miR-200 family.

The disruption of glucose metabolism, insulin signaling, lipid metabolism, and the inflammatory state is directly linked to the development of oxidative stress associated with the obese phenotype (Kilic et al., 2016) (as summarized in Table 2). The continued propagation of these harmful cycles causes positive feedback on cellular pathways that generate inflammatory markers, ROS, pro-apoptotic factors, and metabolic pathway intermediates. These intermediates are then able to cross the BBB and cause oxidative damage to neuronal tissue (Roediger and Armati, 2003; Salim, 2017). Increased oxidative damage in the obese state can encourage the development of neuronal disorders characterized by neurodegeneration as well as cognitive decline later in life (Kim G.H. et al., 2015). The role of miRNAs in the proliferation of obese phenotype associated oxidative stress that causes neurodegenerative diseases cannot be overlooked, as there is evidence to suggest that these molecules do play a role. Aberrant levels of microRNAs cause these homeostatic functions to be disturbed, and further continues the progression of oxidative and inflammatory damage on neuronal tissue.

**TABLE 2:** Summary of mechanistic action of miRNAs in obesity associated cognitive decline and neurodegenerative disorders.

miRNA	Pathway/Mechanism in obesity and metabolic homeostasis	Pathway/Mechanism in cognitive decline and neurodegenerative disorders	References
miR-29a	Insulin resistance Lipid metabolism Glucose homeostasis	Neuronal mitochondrial function Neuroinflammation Aβ deposition	Li et al., 2017; Strosznajder et al., 2021
miR-34a	Inflammation Energy homeostasis	Aβ deposition Tau hyperphosphorylation Neuronal cell death	Ho et al., 1988; Choi et al., 2013; Chua and Tang, 2019; Pan et al., 2019
miR-200 family	Insulin resistance Inflammation	Neuronal apoptosis Aβ deposition α-synuclein aggregation DNA repair	Magenta et al., 2017; Zhang et al., 2017; Fu et al., 2019
miR-26a	Decreased insulin sensitivity Hyperinsulinemia	Tau hyperphosphorylation α-synuclein aggregation Hyperphosphorylation, Lewy Bodies Neurite outgrowth	Li and Sun, 2013; Su et al., 2019; Liu et al., 2020; Xu et al., 2020
miR-425	Apoptosis as a result of lactate build-up Lipogenesis	Tau hyperphosphorylation Phosphorylated AKT survival pathway	Qi et al., 2019; de la Rocha et al., 2020; Luo et al., 2020; Yuan et al., 2020
miR-33	Lipid metabolism Cholesterol homeostasis Fatty acid metabolism HDL Generation	Neuroinflammation Cell Apoptosis Aβ deposition	Cirera-Salinas et al., 2012; Wang and Smith, 2014; Jaouen and Gascon, 2016; Wang et al., 2020
miR-21	Adipogenesis Insulin sensitivity PI3K-AKT pathway	Tau aggregation PIP3 signaling pathway	Zhang et al., 2006a, b; Pal et al., 2012; Kang et al., 2013
miR-144	Insulin signaling Lipid accumulation Adipogenesis Glucose Metabolism Cholesterol metabolism	Aβ deposition Urea Cycle	Chiang et al., 2007; Karolina et al., 2011; Cheng et al., 2013; Shen et al., 2018

Although there is evidence to suggest that miRNAs play important functions in the development of oxidative stress in the obese state that directly impacts neuronal tissue, more research is required to determine the exact role of where these miRNAs play their part. The eight miRNAs that this review investigates did show therapeutic potential as possible targets of treatment. However, the mechanism in which this would be done remains to be further uncovered. In order to investigate the efficacy of miRNAs as therapeutic targets, more defined mechanisms need to be determined. The viability of dietary intervention in the role of improving health outcomes related to obesity and neurodegenerative disorders remains to be important. Increased research in this area could lead to new treatment alternatives that can impact patients worldwide and use miRNA technologies in order to inhibit the progression of the obese state and possible development of neurodegenerative disorders associated with resulting cellular and tissue specific phenotypic alterations.

## Author Contributions

KS: conceptualization. HL and KS: validation and project administration. MP, AK, JW, SL, SP, and DP: writing—original draft preparation. HL: writing—review and editing. KS: supervision and funding. All authors read and agreed to the published version of the manuscript.

## Conflict of Interest

The authors declare that the research was conducted in the absence of any commercial or financial relationships that could be construed as a potential conflict of interest.

## Publisher’s Note

All claims expressed in this article are solely those of the authors and do not necessarily represent those of their affiliated organizations, or those of the publisher, the editors and the reviewers. Any product that may be evaluated in this article, or claim that may be made by its manufacturer, is not guaranteed or endorsed by the publisher.
